# Supplementary material for: Association of Impaired Fasting Glucose and Diabetes with SARS-CoV-2 Spike Antibody Titers after the BNT162b2 Vaccine among Health Care Workers in a Tertiary Hospital in Japan
Source: Vaccines (Basel). 2022 May 13;10(5):776. doi: 10.3390/vaccines10050776 (PMC9148122; doi:10.3390/vaccines10050776)
Supplement: Supplementary file 1 [file vaccines-10-00776-s001.zip › vaccines-1689821-supplementary.pdf]

vaccines

MDPI

Table S1. Multivariable adjusted estimated geometric means (GMT) (95% CI) and ratio of mean (95% CI) of SARS-Cov-2 spike antibody titers according to the glycemic status defined as WHO criteria *.

	**SARS-CoV-2 Spike IgG Antibodies**	
	**GMT (95% CI)**	**Ratio of Mean (95% CI)**
Normoglycemia	5481 (5254–5718)	1.00 (Reference)
IFG	3912 (2244–6819)	0.71 (0.41–1.25)
Diabetes	3418 (2395–4876)	**0.62 (0.43–0.89)**
p § trend		**<0.001**

IFG, impaired fasting glucose; WHO, World Health Organization; FPG, fasting plasma glucose; CI, confidence interval. Values in bold show statistical significance. Model was adjusted for age (year, continuous), sex, BMI (kg/m^2, continuous), cigarette smoking (yes or no), alcohol drinking (non-drinker, occasional drinker, <1 go/day, or ≥1 go/day), hypertension (yes or no), and days after the second vaccination (days, continuous). * Normoglycemia was defined as <110 mg/dl, IFG was defined as FPG 110–125 mg/dl, and diabetes was defined as FPG ≥126 mg/dl or being under medical care for diabetes. § Based on linear regression analysis, assigning ordinal numbers to the normoglycemia, IFG, and diabetes status.
